# Detection of Viral Nucleic Acid in Specimens Spotted on Commercial Filter Papers: A Review and Meta-Analysis

**DOI:** 10.3390/v18060630

**Published:** 2026-05-30

**Authors:** Betsy Armenta-Leyva, Berenice Munguía-Ramírez, Brad Kuennen, Yanqi Zhang, Luis G. Giménez-Lirola, Jeffrey J. Zimmerman

**Affiliations:** 1 Department of Veterinary Diagnostic and Production Animal Medicine, College of Veterinary Medicine, Iowa State University, Ames, IA 50011, USA; bmunguia@iastate.edu (B.M.-R.); luisggl@iastate.edu (L.G.G.-L.); jjzimm@iastate.edu (J.J.Z.); 2 University Library, Iowa State University, Ames, IA 50011, USA; kuennen@iastate.edu; 3 Department of Statistics, College of Liberal Arts and Sciences, Iowa State University, Ames, IA 50011, USA; zyq1998@iastate.edu

**Keywords:** filter paper, virus, PCR, dried blood spots, viral detection

## Abstract

Filter paper-based sampling has been widely used for the collection, transport, and storage of biological samples. This review and meta-analysis aggregated the performance of commercial filter paper matrices for nucleic acid detection across human and veterinary viral pathogens. The review was conducted according to PRISMA guidelines using PubMed^®^, Web of Science^®^, and Scopus™ databases. Using eligible studies, nucleic acid detection rates were calculated as the number of PCR-positive filter paper samples divided by the total number of expected positive sampling units, based on direct testing or experimental design. Detection rates were analyzed using a multilevel meta-analysis of proportions with nested random effects to account for clustering within studies. A total of 145 studies representing 39 filter paper types were included. Cellulose-based matrices, particularly Whatman^®^ and FTA™ products, predominated in the literature, although polyester and glass fiber substrates were also represented. Detection rates varied widely by filter paper type (46.1% to 97.0%) and virus target (63.7% to 92.8%). Experimental conditions, including storage temperature, drying time, and humidity, were inconsistently reported across studies, but the findings indicated that filter paper composition and experimental conditions influenced viral nucleic acid recovery and detection. Overall, this review showed that the recovery and detection of viral nucleic acid from filter paper is variable. The review also highlighted the need for experimental designs providing rigorous comparisons of filter paper performance over a range of conditions.

## 1. Introduction

Filter paper is an efficient, low-cost option for collecting, transporting, and storing a variety of test specimens, including dried blood spots (DBS), biofluids, stool, tissue impression smears, environmental samples, and others [1,2,3,4,5]. Early medical applications capitalized on the capacity of filter paper to stabilize and transport biological material, most notably dried blood spots for antibody testing and newborn metabolic screening [6,7]. While its use in antibody testing remains relevant [8], recent work in human and veterinary medicine has increasingly focused on nucleic acid-based testing for a variety of pathogens, including human immunodeficiency virus (HIV), porcine reproductive and respiratory syndrome virus (PRRSV) in pigs, and astrovirus-1 in specific pathogen-free (SPF) laboratory animal colonies [9,10,11].

Filter papers vary in their composition and physical properties. For example, cellulose-based papers, e.g., Whatman^®^ (Millipore Sigma, Burlington, MA, USA), provide mechanical robustness and nucleic acid entrapment, but variability in pore structure can influence nucleic acid recovery yields [12,13]. Other materials, e.g., polymer-based filter papers, offer greater durability during handling and processing. For instance, polyester filter paper retains structural integrity when moistened compared to cotton or glass fiber matrices [12]. Filter papers may also contain chemically incorporated reagents that influence nucleic acid stability. Chemically treated substrates, such as Flinders Technology Associates (FTA) cards (Millipore Sigma), incorporate reagents that promote cell lysis and nucleic acid stabilization, which may improve nucleic acid purity while introducing trade-offs in nucleic acid elution efficiency [12,14]. Collectively, these characteristics may influence filter paper performance in molecular diagnostic applications.

Although filter papers are diverse, direct comparisons of their PCR performance characteristics are uncommon in the peer-reviewed literature. Where such comparisons are available, studies often differ in experimental design, specimen type, pathogen target, assay platform, storage conditions, and outcome metrics, thereby complicating the selection of the appropriate filter paper matrix for a specific application. Accordingly, the objective of this review was to evaluate differences in the rate of nucleic acid detection across specimen types as a function of the filter paper matrix.

## 2. Methods

### 2.1. Study Overview

The review was conducted according to the Preferred Reporting Items for Systematic Reviews and Meta-Analyses (PRISMA) guidelines, as described in a registered protocol (Open Science Framework registration: RQBC4) [15].

### 2.2. Search Methodology

**Figure 1:**
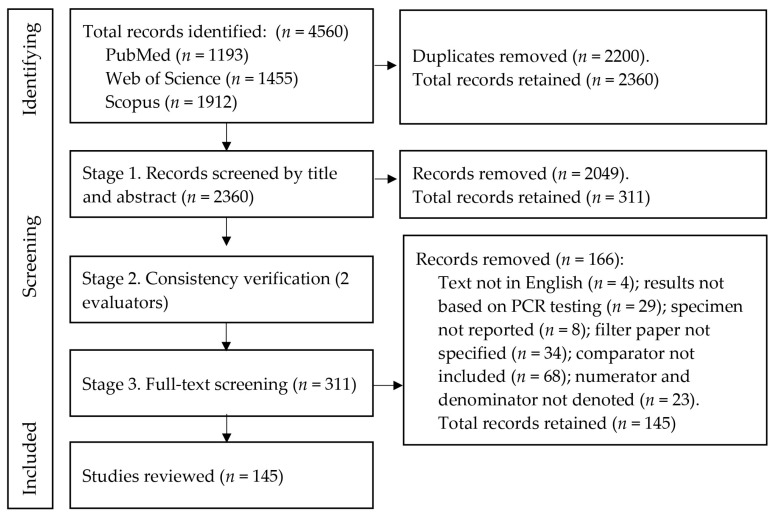
Flow diagram of the search strategy and process for selecting studies to be included in the analyses. Adapted from PRISMA 2020 statement [19].

The literature search was performed in three databases: PubMed^®^ [16], Web of Science^®^ (Clarivate™), and Scopus™ (Elsevier™), accessed through their respective interfaces using a broad search algorithm that included terms for viruses, filter paper types/brands, and PCR testing (Table 1). As described in Figure 1, this process resulted in the identification of 2360 records after deduplication.

### 2.3. Inclusion Criteria

Peer-reviewed publications were evaluated for inclusion based on experimental design and analysis following the PICO (Population, Intervention, Comparison, and Outcome) guidelines. In brief, publications were included if they were published in English, clearly described the specimen applied to a well-characterized filter paper (manufacturer, grade, and composition), the PCR methodology used (gel-based or real-time), and reported detection rates as the proportion of PCR-positive filter paper samples (numerator) over the total number of reference samples (denominator).

Publications recovered from the search process (*n* = 2360) were screened in three stages (Figure 1). (1) publications were screened for relevance based on titles and abstracts using Rayyan software (v.1.6.0) [17] based on a single relevance question: “Did the study report viral detection using filter paper-based sampling?” Records not meeting this criterion were excluded, leaving 311 texts for full evaluation.

(2) Full texts were screened using a structured eligibility questionnaire implemented in DistillerSR (https://www.distillersr.com) [18]. To establish consistency in the full text evaluation process, two evaluators independently evaluated a subset of publications (*n* = 63). Discordant evaluations were discussed and used to establish uniformity in the review process. (3) The remaining potentially eligible studies (*n* = 248) were evaluated by one screener (BAL).

### 2.4. Data Collected for Analysis

Descriptive data collected from studies included in the analyses included filter paper descriptors (manufacturer, grade, and composition), PCR protocol, virus identity (taxonomy and genomic classification), host species, and specimen type. Storage conditions (drying time, temperature, humidity, container type) were collected if available, but due to substantial heterogeneity, storage conditions were not included in the analysis.

A number of variations in experimental design were observed, e.g., independent vs. repeated sampling and known vs. unknown infection status of the sampling unit(s). Therefore, to establish a basis for comparison, detection rates were standardized across studies as the number of PCR-positive filter paper samples divided by the total number of expected positive sampling units, as described in Table 2.

### 2.5. Meta Analyses

Statistical analyses were conducted in RStudio v.4.5.3 [20]. Detection rate data extracted from eligible studies (*n* = 145) were analyzed to estimate pooled viral nucleic acid detection rates. Proportions were logit-transformed and analyzed using a multilevel meta-analysis of proportions (metafor package v.4.8-0) [21]. A nested random-effects structure was specified to account for clustering of observations within studies, thereby allowing multiple observations per study while modeling within-study correlation. Models were fit using restricted maximum likelihood estimation and observations were weighted by inverse variance. Model estimates were obtained on the logit scale and back-transformed to proportions to generate pooled detection rates with corresponding 95% confidence intervals (95% CI).

Two separate models were fitted using the same specifications, differing only in the categorical variable included as a fixed effect. In the first model, filter paper type (defined by brand and, when available, grade) was included as a fixed effect to estimate pooled detection rates across paper types. In the second model, virus target was included as a fixed effect to estimate pooled detection rates by virus. For the virus-level analysis, only viruses analyzed in at least three independent studies were included.

**Table 2:** Calculation of rate of detection of nucleic acid (NA) from filter papers based on experimental design ^1^.

Sampling Design	Infection Status of Sampling Unit(s)	Types of Comparisons (Numerator, Denominator)	Examples
A. Samples collected from sampling unit(s) at one point in time, i.e., observations are independent.	a. Unknown	1. Numerator based on testing filter paper inoculated with a defined specimen from a defined sampling unit. Denominator (total expected positive) is based on directly testing the same biological specimen from the same sampling unit(s).	Numerator—dried blood spots from individual persons tested for HIV. Denominator—whole blood collected from the same individual and tested for HIV [22].
		2. Numerator based on testing filter paper inoculated with a defined specimen from a defined sampling unit. Denominator (total expected positive) based on directly testing a different specimen from the same sampling unit(s).	Numerator—dried oral mucosal brush spots from individual humans tested for HPV. Denominator—tumor biopsy samples collected from the same individual [23].
	b. Known	3. Numerator based on testing filter paper inoculated with a defined specimen. Denominator (total expected positive) implicit in the experimental design.	Numerator—dried oral mucosa swab spots tested for FMDV from individual inoculated cattle. Denominator implicit in experimental design, i.e., number of inoculated animals [24].
B. Samples collected from sampling unit(s) repeatedly over time, i.e., observations are correlated	c. Unknown	4. Same as 1 (above)	Numerator—dried blood spots from individual pigs tested for PRRSV. Denominator—whole blood collected from pigs periodically (28 days) and tested for PRRSV [25].
	d. Known	5. Same as 3 (above)	Numerator—dried brain homogenate spots tested for West Nile virus from individual inoculated mice and tested periodically for 90 days [26]. Denominator implicit in experimental design, i.e., number of inoculated mice.

^1^ In all cases, the numerator was based on the detection of nucleic acid in filter paper inoculated with the specimen of interest and the denominator was based on the total number of expected positive sampling units.

## 3. Results

**Figure 2:**
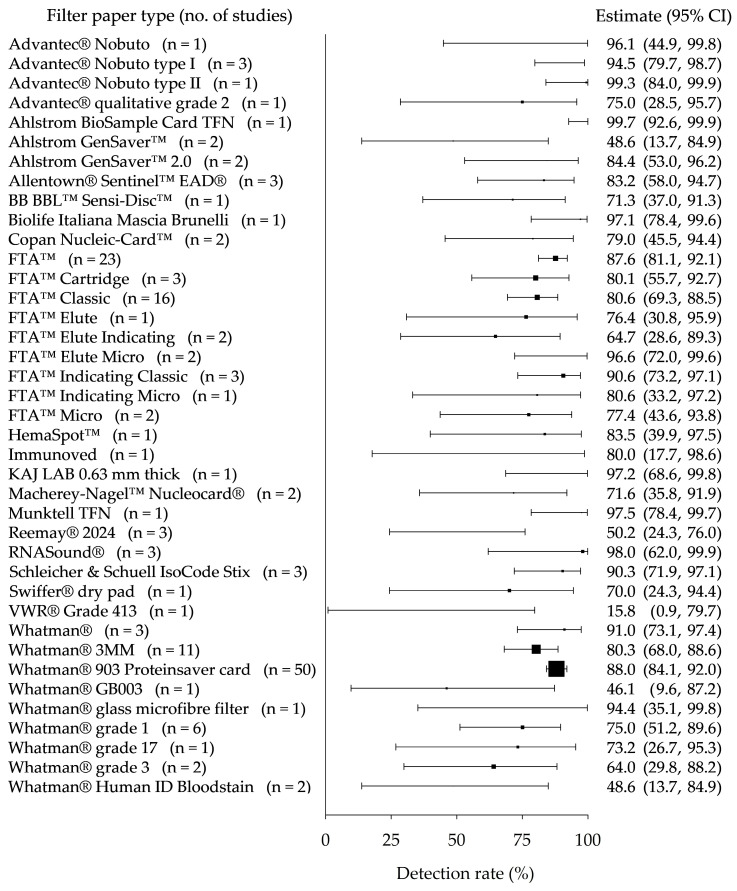
Forest plot of viral nucleic acid detection rates by filter paper type based on a multilevel meta-analysis of proportions. Each symbol represents the pooled detection rate, with bars denoting the 95% confidence interval. Advantec^®^ Nobuto [27]; Advantec^®^ Nobuto Type I [28,29,30]; Advantec^®^ Nobuto type II [31]; Advantec ^®^ Nobuto qualitative grade 2 [25]; Ahlstrom BioSample Card TFN [32]; Ahlstrom GenSaver™ [24,33]; Ahlstrom GenSaver™ 2.0 [24,33]; Allentown^®^ Sentinel™ EAD^®^ [11,34,35]; BB BBL™ Sensi-Disc™ [36]; Biolife Italiana Mascia Brunelli [37]; Copan Nucleic-Card™ [24,33]; FTA™ [9,36,38,39,40,41,42,43,44,45,46,47,48,49,50,51,52,53,54,55,56,57,58]; FTA™ Cartridge [59,60,61]; FTA™ Classic [24,33,62,63,64,65,66,67,68,69,70,71,72,73,74,75]; FTA™ Elute [76]; FTA™ Elute Indicating [23,33]; FTA™ Elute Micro [77,78]; FTA™ Indicating Classic [79,80,81]; FTA™ Indicating Micro [82]; FTA™ Micro [83,84]; HemaSpot™ [85]; Immunoved [86]; KAJ LAB 0.63 mm thick [87]; Macherey-Nagel™ Nucleocard^®^ [24,33]; Munktell TFN [88]; Reemay^®^ grade 2024: [35,89,90]; RNASound^®^ [82]; Schleicher & Schuell IsoCodeStix: [91,92,93]; Swiffer^®^ dry pad [94]; VWR^®^ Grade 413 [24]; Whatman^®^ [92,93,95]; Whatman^®^ 3MM [96,97,98,99,100,101,102,103,104,105,106]; Whatman^®^ 903 Proteinsaver card [2,3,21,107,108,109,110,111,112,113,114,115,116,117,118,119,120,121,122,123,124,125,126,127,128,129,130,131,132,133,134,135,136,137,138,139,140,141,142,143,144,145,146,147,148,149,150,151,152,153]; Whatman^®^ GB003 [154]; Whatman^®^ glass microfibre filter [155]; Whatman^®^ grade 1 [24,25,156,157,158,159]; Whatman^®^ grade 17 [160]; Whatman^®^ grade 3 [161,162]; Whatman^®^ Human ID Bloodstain: [24,33].

The first statistical model used detection rate data from 145 studies on 39 filter paper types (Figure 2). The majority of filter papers were cellulose-based, including widely used commercial brands, e.g., Whatman^®^, FTA™, Advantec^®^, Ahlstrom, and others. A small number of studies employed alternative materials. Polyester-based materials included Reemay^®^, Allentown^®^ Sentinel™, and Swiffer^®^. Glass fiber formats were represented by Immunoved, and Whatman^®^ glass microfibre filters. Several papers used materials of uncertain composition, e.g., Schleicher & Schuell IsoCode Stix, Copan Nucleic-Card™, and KAJ LAB 0.63 mm thick cards.

Experimental design was variable among studies. In particular, publications inconsistently reported filter paper drying times, temperature, relative humidity, and, when included, descriptions were frequently incomplete. When reported, these parameters were frequently incompletely described. Overall, 60% (87 of 145 studies) reported one or more parameters and no single parameter was consistently documented in all publications (Table 3). A complete list of the studies and their basic elements of experimental design is given in Appendix A (Table A1).

**Figure 3:**
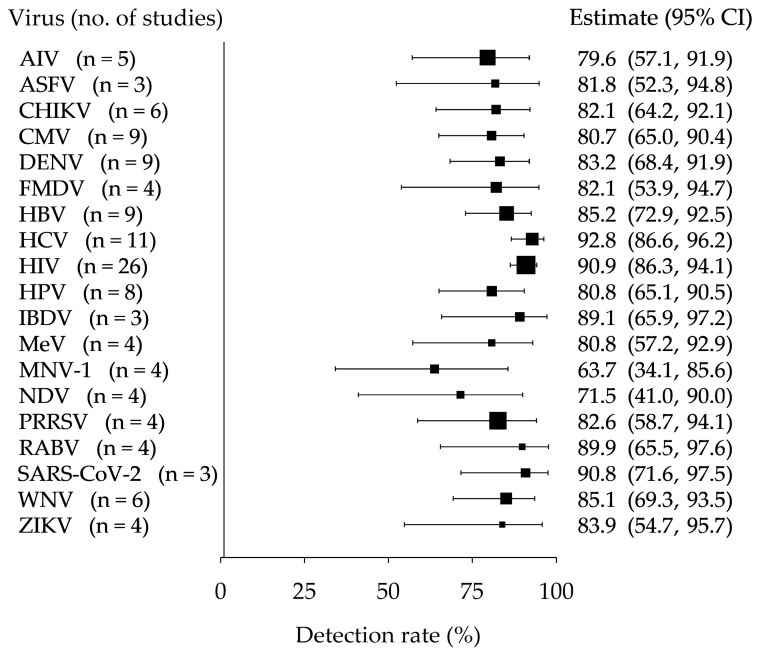
Forest plot of nucleic acid detection rates by virus, based on a multilevel meta-analysis of proportions (≥3 studies per virus). Each symbol represents the pooled detection rate, with bars denoting the 95% confidence interval. See list of abbreviations. AIV [38,46,65,72,94]; ASFV [33,103,104]; CHIKV [41,44,96,102,115,126]; CMV [76,110,111,114,120,134,143,144,148]; DENV [30,45,58,68,83,107,115,126,149]; FMDV [24,49,67,69]; HBV [71,85,100,113,117,119,132,139,145]; HCV [62,88,112,122,124,139,140,142,146,155,156]; HIV [22,32,77,91,93,108,109,116,118,121,122,123,125,127,128,129,130,131,133,136,137,138,147,152,153,162]; HPV [23,37,59,60,61,78,97,105]; IBVD [50,51,55]; MeV [2,79,141,161]; MNV-1 [34,35,89,90]; NDV [52,53,72,159]; PRRSV [9,25,43,49]; RABV [3,54,63,150]; SARS-CoV-2 [66,87,106]; WNV [26,44,56,64,82,154]; ZIKV [45,95,115,126].

The second statistical model estimated detection rates for specific viruses (*n* = 19) in cases where ≥3 independent studies were available (Figure 3). The number of contributing studies varied across viruses, with the greatest representation observed for human immunodeficiency virus (HIV), hepatitis C virus (HCV), and cytomegalovirus (CMV). Overall, mean detection rate estimates ranged from 63.7 to 92.8%.

## 4. Discussion

This review provided an overview of the use of filter papers in diagnostic medicine, with a focus on the recovery and detection of viral nucleic acids. Reflecting their long use and commercial availability, FTA™ and Whatman^®^ samplers were predominant in the published studies. Guthrie’s original newborn screening cards (Guthrie cards), introduced in the early 1960s, were prepared on Whatman No. 3 filter paper [163]. Indeed, the use of filter paper in sample collection is often considered to have begun with the collection of dried blood spots (DBS) from neonates on Guthrie cards for phenylketonuria screening [6], but its use in sampling actually began earlier. For example, Boyd and Hanson (1958) demonstrated that viable Newcastle disease virus could be recovered from paper discs stored for 20 days at 20 °C [164]. Soon thereafter, Sadun et al. (1961) demonstrated that DBS on filter paper sent through the mail were effective for schistosomiasis (*Schistosoma japonicum*) fluorescent antibody testing [165]. Later, with the development of molecular diagnostics, methods were developed for the recovery of viral nucleic acid from filter paper. Early studies showed that samples containing hepatitis B virus, HIV proviral DNA, infectious bursal disease virus, and hemorrhagic enteritis virus could be collected on paper and viral nucleic acids extracted and amplified in the laboratory [100,152,166]. Filter paper sampling remains practical and relevant to diagnostic medicine, with recent studies demonstrating its utility for SARS-CoV-2 detection [66,87,106].

The mechanism by which nucleic acids are preserved on paper matrices reflects “dry-state stabilization”. That is, the structure and biological function of molecules (proteins, enzymes, and nucleic acids) are preserved when stored in a dry or solid state [167]. Essentially, the immobilization of biological material in a dehydrated matrix suppresses enzymatic activity and microbial degradation, thereby allowing nucleic acids to remain intact, recoverable, and amplifiable in the laboratory [168]. Consequently, filter papers function as both sample transport and storage matrices, providing a simple yet effective means of preserving DNA and RNA without reliance on cold-chain infrastructure. Efforts to enhance this effect have included saturating or embedding stabilizing and lytic reagents in filter papers for the purpose of inactivating pathogens yet preserving their nucleic acids. Thus, FTA™ cards include chemical components designed to capture and preserve nucleic acids [169]. Alternatively, Wollants et al. (2004) demonstrated that chromatography paper strips pretreated with sodium dodecyl sulfate and EDTA could safely collect and transport norovirus RNA from stool samples, with amplifiable RNA detectable for up to two months from paper samplers held at room temperature [5].

While the principle of dry-state stabilization is well established, the practical utility of filter paper sampling for PCR-based detection ultimately depends on the preservation and recovery of amplifiable nucleic acids under field conditions. PCR-based detection outcomes, in turn reflect both the ability of filter papers to stabilize nucleic acids and the recoverability of nucleic acids from the matrix. These characteristics reflect (1) composition of the filter paper, (2) storage conditions, and (3) specimen.

Filter papers are manufactured from a variety of materials and in various formats, although cellulose and cellulose-treated matrices are the most frequently described in the literature. Published comparisons among filter paper types are limited and the performance characteristics attributable to specific materials and formats are not well characterized. Regardless, recent work demonstrated that filter paper composition can influence sampler performance. For example, Armenta-Leyva et al. (2025), showed differences in the recovery of viral RNA from filter paper types, with polyester-based materials outperforming cellulose-based papers and, under optimized conditions, achieving recovery comparable to direct sample testing [170]. Similarly, other studies reported that polyester-based matrices enhanced the capture of target in laboratory animal environmental PCR monitoring [171].

Equally impactful on target recovery are storage conditions, e.g., drying conditions, temperature, relative humidity, and storage time [14,22,172,173]. For instance, Sakai et al., (2014) showed that rabies viral RNA stored on FTA^®^ cards remained stable for months at −20 °C or −80 °C, but degraded within weeks at 4 °C or room temperature [174]. Similarly, Armenta-Leyva et al. (2026) demonstrated that PRRSV and PEDV RNA inoculated onto polyester and cellulose papers remained stable for 28 days under low humidity (<20%) but relative humidity levels ≥ 40% accelerated decay [172]. Importantly, preservation of detectable nucleic acid on filter paper matrices should not be interpreted as evidence of preserved or inactivated pathogen infectivity. The studies in this review primarily evaluated PCR-based molecular detectability rather than residual infectivity following drying, storage, transport, or handling. Although certain chemically treated matrices, such as FTA™ cards, are designed to inactivate pathogens while stabilizing nucleic acids, infectivity outcomes were outside of the scope of the present review. Consequently, the biosafety implications associated with handling, transport, and storage of spotted biological materials remain an important area for future investigation.

To a lesser extent, the specific specimen inoculated onto the filter paper is a source of variability. Even within blood samples, detection rates are affected by the fraction selected, e.g., buffy coat preparations yielded more PCR-positive samples for *Trypanosoma brucei* than DBS [175]. Similarly, Smit et al. (2014) observed that DBS prepared from whole blood often produced lower HIV antibody titers than serum [176]. The test analyte may also play a role: nucleic acids tend to remain stable longer than proteins [177].

Previous systematic reviews generally reported diagnostic sensitivities and specificities of 90 to 100% for major human pathogens, e.g., HIV, HBV, and HCV [176,178,179,180], but these reviews primarily focused on DBS as a specimen type and included a limited number of filter paper types. The current review included both human and veterinary pathogens and all filter paper types reported in the peer-reviewed literature. A meta-analysis based on filter paper types (first statistical model), found that detection rates ranged from 46.1% to 97.0%; a meta-analysis based on virus target (second statistical model) found that detection rates ranged from 63.7% to 92.8%. The greater range in detection rates shown in our analyses likely reflects the aggregate effect of substrate composition, experimental design, storage conditions, extraction procedures, and target-specific factors, many of which were inconsistently reported across studies. Importantly, given the heterogeneity in study design and reporting practices across the included literature, the present review synthesized PCR-based detection using binary detection outcomes because this represented the most consistently reported endpoint across publications. Quantitative measures such as quantification cycles (Cq) values, dilution series performance, and limits of detection were variably reported and frequently unavailable at the individual-sample level, limiting standardized comparison of quantitative recovery across methodologies. Despite this, substantial variability in detection rates was still observed across filter paper types and virus targets, suggesting that the selected endpoint retained sufficient discriminatory capacity to capture meaningful differences. Notably, even widely used substrates such as Whatman^®^ and FTA™ exhibited broad ranges in detection rates. Nevertheless, binary outcomes do not fully characterize analytical sensitivity or quantitative recovery efficiency, emphasizing the need for more standardized reporting practices.

In summary, the diagnostic use of filter paper in sampling, transport, and storage remains compelling because of its simplicity, reliability, and cost-effectiveness for a variety of analytes. However, our meta-analyses suggest that more exacting diagnostic performance estimates and greater methodological standardization may be required for filter paper to fully realize its potential in clinical and field settings.

## Figures and Tables

**Table 1:** Search terms used in the review.

Variable	Description
1	“virus*”[Title/Abstract] OR “viruses”[MeSH Terms] OR “viral”[Title/Abstract] OR “dna viral”[MeSH Terms] OR “rna viral”[MeSH Terms]
2	“filter paper*”[tiab] OR Whatman[tiab] OR FTA[tiab] OR Flinders[tiab] OR Reemay[tiab] OR “Sentinel EAD”[tiab:~0] OR “Schleicher Schuell”[tiab:~2] OR Guthrie[tiab] OR “nucleic acid cards”[tiab:~1] OR “exhaust air dust”[tiab] OR “saver card*”[tiab] OR “filter media”[tiab] OR “toyo roshi kaisha”[tiab:~1] OR ISOCODE[tiab] OR Munktell[tiab] OR Ahlstrom[tiab] OR Nobuto[tiab] OR “environmental sampl*”[tiab] OR “dried spots”[tiab:~1]
3	PCR[tiab] OR “polymerase chain reaction”[tiab] OR polymerase chain reaction[MeSH Terms] OR “RT-PCR”[tiab] OR “RT-qPCR”[tiab] OR “molecular method*”[tiab]
4	#1 AND #2 AND #3
1	TS = (virus* OR viral)
2	TS = (“filter paper*” OR Whatman OR FTA OR Flinders OR Reemay OR (Sentinel NEAR/0 EAD) OR (Schleicher NEAR/2 Schuell) OR Guthrie OR (“nucleic acid” NEAR/1 (card OR cards)) OR “exhaust air dust” OR “saver card*” OR “filter media” OR “toyo roshi kaisha” OR ISOCODE OR Munktell OR Ahlstrom OR Nobuto OR “environmental sampl*” OR (dried NEAR/1 spots))
3	TS = (PCR OR “polymerase chain reaction” OR “RT-PCR” OR “RT-qPCR” OR “molecular method*”)
4	#1 AND #2 AND #3
1	TITLE-ABS-KEY (virus* OR viral)
2	TITLE-ABS-KEY (“filter paper*” OR Whatman OR FTA OR Flinders OR Reemay OR (Sentinel W/0 EAD) OR (Schleicher W/2 Schuell) OR Guthrie OR (“nucleic acid” W/1 (card OR cards)) OR “exhaust air dust” OR “saver card*” OR “filter media” OR “toyo roshi kaisha” OR ISOCODE OR Munktell OR Ahlstrom OR Nobuto OR “environmental sampl*” OR (dried W/1 spots))
3	TITLE-ABS-KEY (PCR OR “polymerase chain reaction” OR “RT-PCR” OR “RT-qPCR” OR “molecular method*”)
4	#1 AND #2 AND #3

**Table 3:** Parameters relevant to the recovery and detection of nucleic acid from filter paper.

Parameters	Studies Reporting	Range	Summary
Drying times	80/145	0.24 to 48 h	Among studies reporting drying times, 80% were between 0.5 and 24 h. Median drying time was 4 h, i.e., one-half of studies reported shorter times.
Drying temperature	59/145	20 to 33 °C	Among studies reporting drying temperature, 80% fell between 21 °C and 29 °C; with a median of 24 °C.
Storage temperature	87/145	−80 to 41 °C	Among studies reporting storage temperature, 80% fell between −35 °C and 37.9 °C; with a median of 23.5 °C.
Storage relative humidity	3/145	53 to 70%	Three studies reported relative humidity during storage.
Storage time	46/145	0 to 2920 d	Among studies reporting storage time, 80% were between 3.6 days and 803.2 days; with median of 56 days.

## Data Availability

The original contributions presented in the study are included in the article, further inquiries can be directed to the corresponding author.

## Abbreviations

The following abbreviations are used in this manuscript:

AIV	Avian influenza virus
AMPV	Avian metapneumovirus
ASFV	African swine fever virus
AstV-1	Astrovirus 1
BCV	Bovine coronavirus
BFV	Barmah forest virus
BHV-1	Bovine herpesvirus 1
BLV	Bovine leukemia virus
BRSV	Bovine respiratory syncytial virus
BVDV	Bovine viral diarrhea virus
CHIKV	Chikungunya virus
CMV	Cytomegalovirus
DBS	Dried blood spots
DENV	Dengue virus
DHBV	Duck hepatitis B virus
DMV	Dolphin Morbillivirus
EBV	Epstein–Barr virus
FAdV	Fowl adenovirus
FMDV	Foot-and-mouth disease virus
FTA	Flinders Technology Associates
HAdV-2	Human adenovirus 2
HAV:	Hepatitis A virus
HBV	Hepatitis B virus
HCV	Hepatitis C virus
HEV	Hepatitis E virus
HIV	Human immunodeficiency virus
HPV	Human papillomavirus
IAV	Influenza A virus
IBDV	Infectious bursal disease virus
IBV	Infectious bronchitis disease virus
IHHNV	Infectious hypodermal and hematopoietic necrosis virus
MDV	Marek’s disease virus
MeV	Measles virus
MHV	Mouse hepatitis virus
MNV	Murine norovirus
NA	Nucleic acid
NDV	Newcastle disease virus
NNV	Nervous necrosis virus
NoV	Norovirus
PCR	Polymerase chain reaction
PICO	Population, intervention, comparison, and outcome.
PPRV	Peste des petits ruminants virus
PRISMA	Preferred reporting items for systematic reviews and meta-analysis
PRRSV	Porcine reproductive and respiratory syndrome virus
PUUV	Puumala hantavirus
RABV	Rabies virus
RHDV2	Rabbit Hemorrhagic disease virus 2
RRV	Ross river virus
RV	Rotavirus
SARS-CoV-2	Severe acute respiratory syndrome coronavirus 2
SIV	Simian immunodeficiency virus
SPF	Specific pathogen free
TBEV	Tick-borne encephalitis virus
TMEV	Theiler’s murine encephalomyelitis virus
USUV	Usutu virus
VEEV	Venezuelan equine encephalitis virus
WNV	West Nile virus
YFV	Yellow fever virus
ZIKV	Zika virus

## Appendix A

**Table A1:** Characteristics of included studies and PCR-based detection rates for filter paper sampling methods.

Virus	Year	Filter Paper	Specimen	Comparison Type	Detection Rate (%) (Pos/Denominator)	Reference
AIV	2011	FTA^™^	Allantoic fluid	2	80.0 (12/15)	[38]
	2012	FTA^™^	Cloacal swab	1	87.0 (20/23)	[46]
	2012	FTA^™^	Oropharyngeal swab	1	100.0 (5/5)	[46]
	2016	FTA^™^ Classic	Allantoic fluid	4	83.3 (5/6)	[65]
	2021	Swiffer^®^ dry pad	Virus culture	5	70.2 (59/84)	[94]
	2022	FTA^™^ Classic	Allantoic fluid	4	75.0 (6/8)	[72]
AMPV	2014	FTA^™^	Virus culture	4	66.7 (4/6)	[39]
ASFV	2007	Whatman^®^ 3MM	Whole blood	1	88.9 (8/9)	[103]
	2013	FTA^™^ Classic		3	100.0 (10/10)	[75]
	2014	Whatman^®^ 3MM		1	65.2 (45/69)	[104]
	2021	FTA^™^ Classic		5	100.0 (4/4)	[33]
	2021	FTA^™^ Indicating		5	100.0 (4/4)	[33]
	2021	Ahlstrom GenSaver^™^		5	100.0 (4/4)	[33]
	2021	Ahlstrom GenSaver^™^ 2.0		5	100.0 (4/4)	[33]
	2021	Whatman^®^ Human ID Bloodstain		5	100.0 (4/4)	[33]
	2021	Copan Nucleic-Card^™^		5	100.0 (4/4)	[33]
	2021	Macherey-Nagel^™^ Nucleocard^®^		5	100.0 (4/4)	[33]
AstV-1	2022	Allentown^®^ Sentinel^™^ EAD^®^	Soiled bedding	2	100.0 (4/4)	[11]
BCV	2014	FTA^™^ Indicating	Nasal swab	3	100.0 (30/30)	[80]
BFV	2017	FTA^™^	Mosquitoes	5	100.0 (4/4)	[45]
BHV-1	2014	FTA^™^ Indicating	Nasal swab	3	92.7 (29/30)	[80]
	2018	FTA^™^	Semen	1	93.2 (234/251)	[57]
BLV	2014	Immunoved	Whole blood	1	80.0 (4/5)	[86]
	2020	Advantec^®^ Nobuto type I	Whole blood	3	97.0 (64/66)	[28]
BRSV	2014	FTA^™^ Indicating	Nasal swab	3	100.0 (30/30)	[80]
BVDV	2001	Whatman^®^ grade 1	Whole blood	1	100.0 (8/8)	[157]
	2014	FTA^™^ Indicating	Nasal swab	3	100.0 (30/30)	[80]
CHIKV	2010	FTA^™^	Mosquito saliva	3	70.0 (21/30)	[44]
	2013	Whatman^®^ 3MM	Whole blood	1	93.2 (68/73)	[96]
	2015	Whatman^®^ 3MM	Serum	1	92.7 (38/41)	[102]
	2020	Whatman^®^ 903 Proteinsaver card	Serum	1	100.0 (3/3)	[126]
	2022	Whatman^®^ 903 Proteinsaver card	Whole blood	1	100.0 (2/2)	[115]
	2025	FTA^™^	Mosquito saliva	3	100.0 (66/66)	[41]
CMV	2008	Whatman^®^ 903 Proteinsaver card	Whole blood	1	81.8 (45/55)	[144]
	2010	Whatman^®^ 903 Proteinsaver card		2	34.4 (11/32)	[114]
	2013	Whatman^®^ 3MM		3	94.3 (33/35)	[101]
	2013	FTA^™^ Elute		1	76.4 (81/106)	[76]
	2015	Whatman^®^ 903 Proteinsaver card		3	100.0 (19/19)	[120]
	2019	Whatman^®^ 903 Proteinsaver card		3	56.3 (58/103)	[148]
	2020	Whatman^®^ 903 Proteinsaver card	Saliva	1	100.0 (42/42)	[134]
	2025	Whatman^®^ 903 Proteinsaver card	Whole blood	1	77.8 (49/63)	[143]
DENV	2005	Advantec^®^ Nobuto type I	Whole blood	1	93.0 (40/43)	[30]
	2016	Whatman^®^ 903 Proteinsaver card		1	88.6 (31/35)	[107]
	2016	Whatman^®^ 903 Proteinsaver card		1	72.9 (35/48)	[149]
	2017	FTA^™^	Mosquitoes	5	100.0 (4/4)	[45]
	2017	FTA^™^ Classic	Mosquito saliva	3	66.7 (20/30)	[68]
	2020	Whatman^®^ 903 Proteinsaver card	Serum	1	100.0 (4/4)	[126]
	2020	FTA^™^ Micro	Serum	1	77.8 (14/18)	[83]
	2022	Whatman^®^ 903 Proteinsaver card	Whole blood	1	100.0 (4/4)	[115]
	2023	FTA^™^	Fly abdomen	3	87.5 (21/24)	[58]
DHBV	2002	Whatman^®^ grade 1	Serum	1	100.0 (22/22)	[158]
DMV	2023	FTA™ Classic	Tissue (brain, kidney, liver, lung, lymph nodes, spleen, tonsils)	1	47.4 (9/19)	[74]
EBV	2024	Whatman^®^ 903 Proteinsaver card	Whole blood	1	38.8 (19/49)	[135]
	2026	FTA^™^ Classic	Whole blood	1	57.1 (12/21)	[73]
FAdV	2007	FTA^™^ Indicating Classic	Liver impression	5	100.0 (6/6)	[81]
FMDV	2008	FTA^™^ Classic	Oral mucosa swab	1	88.9 (24/27)	[69]
	2013	FTA^™^	Oral mucosa swab	3	100.0 (15/15)	[49]
	2014	FTA^™^ Classic	Tissue impression smear	3	100.0 (8/8)	[67]
	2022	Ahlstrom GenSaver^™^	Virus culture	3	0.0 (0/7)	[24]
	2022	Ahlstrom GenSaver^™^ 2.0		3	71.4 (5/7)	[24]
	2022	FTA^™^ Classic		3	71.4 (5/7)	[24]
	2022	Whatman^®^ Human ID Bloodstain		3	0.0 (0/7)	[24]
	2022	Copan Nucleic-Card^™^		3	71.4 (5/7)	[24]
	2022	Macherey-Nagel^™^ Nucleocard^®^		3	42.9 (3/7)	[24]
	2022	Whatman^®^ grade 1		3	0.0 (0/7)	[24]
	2022	VWR^®^ Grade 413		3	0.0 (0/7)	[24]
HAdV-2	2019	Whatman^®^ 3MM	Virus culture	5	100.0 (4/4)	[99]
HAV	2009	FTA^™^	Serum	1	92.3 (24/26)	[42]
	2019	Whatman^®^ 3MM	Virus culture	5	100.0 (2/2)	[99]
HBV	1992	Whatman^®^ 3MM	Whole blood	1	71.7 (43/60)	[100]
	2004	Whatman^®^ 903 Proteinsaver card		1	87.8 (72/82)	[119]
	2013	Whatman^®^ 903 Proteinsaver card		1	93.0 (93/100)	[139]
	2016	Whatman^®^ 903 Proteinsaver card		1	88.5 (23/26)	[145]
	2017	FTA^™^ Classic	Oral fluid	1	100.0 (16/16)	[71]
	2022	Whatman^®^ 903 Proteinsaver card	Whole blood	1	93.7 (59/63)	[113]
	2022	Whatman^®^ 903 Proteinsaver card		1	48.4 (31/64)	[132]
	2022	Whatman^®^ 903 Proteinsaver card		1	95.3 (81/85)	[117]
	2024	HemaSpot^™^		1	83.6 (56/67)	[85]
HCV	1998	Whatman^®^ glass microfibre filter	Serum	1	100.0 (8/8)	[155]
	2012	Whatman^®^ 903 Proteinsaver card	Whole blood	1	100.0 (38/38)	[140]
	2012	Whatman^®^ 903 Proteinsaver card		1	100.0 (57/57)	[112]
	2013	Whatman^®^ 903 Proteinsaver card		1	100.0 (100/100)	[139]
	2014	Whatman^®^ 903 Proteinsaver card		1	92.3 (48/52)	[146]
	2014	Whatman^®^ 903 Proteinsaver card		3	90.0 (18/20)	[111]
	2016	Whatman^®^ 903 Proteinsaver card		1	79.5 (35/44)	[124]
	2018	Whatman^®^ grade 1		1	55.6 (5/9)	[156]
	2018	Whatman^®^ 903 Proteinsaver card		1	100.0 (42/42)	[142]
	2018	Munktell TFN		1	97.5 (79/81)	[88]
	2021	Whatman^®^ 903 Proteinsaver card		1	89.3 (92/103)	[122]
	2022	FTA^™^ Classic	Plasma	5	68.2 (15/22)	[62]
HEV	2009	Whatman^®^	Whole blood	1	83.8 (31/37)	[92]
	2009	Schleicher & Schuell IsoCode Stix	Whole blood	1	95.1 (39/41)	[92]
HIV	1991	Whatman^®^ 903 Proteinsaver card	Whole blood	3	95.7 (66/69)	[116]
	1992	Whatman^®^ 903 Proteinsaver card		3	100.0 (13/13)	[152]
	1994	Whatman^®^ 903 Proteinsaver card		1	94.4 (17/18)	[131]
	2005	Schleicher and Schuell IsoCode Stix		1	84.0 (42/50)	[93]
	2005	Whatman^®^		1	94.0 (47/50)	[93]
	2005	Whatman^®^ 903 Proteinsaver card		1	85.7 (36/42)	[22]
	2007	Whatman^®^ grade 3		3	78.9 (30/38)	[162]
	2007	Whatman^®^ 903 Proteinsaver card		1	86.7 (26/30)	[108]
	2007	Whatman^®^ 903 Proteinsaver card		1	92.1 (35/38)	[125]
	2008	Schleicher & Schuell IsoCode Stix		1	94.7 (18/19)	[91]
	2009	Whatman^®^ 903 Proteinsaver card		1	100.0 (56/56)	[121]
	2009	Whatman^®^ 903 Proteinsaver card		3	83.7 (36/43)	[138]
	2010	FTA™ Elute Micro		1	100.0 (8/8)	[77]
	2010	Whatman^®^ 903 Proteinsaver card		1	100.0 (12/12)	[128]
	2010	Whatman^®^ 903 Proteinsaver card		1	92.6 (327/353)	[147]
	2010	Whatman^®^ 903 Proteinsaver card		1	88.2 (97/110)	[109]
	2010	Whatman^®^ 903 Proteinsaver card		3	98.3 (59/60)	[130]
	2011	Whatman^®^ 903 Proteinsaver card		3	85.6 (231/270)	[118]
	2011	Whatman^®^ 903 Proteinsaver card		1	88.2 (75/85)	[136]
	2012	Whatman^®^ 903 Proteinsaver card		1	100.0 (412/412)	[133]
	2013	Whatman^®^ 903 Proteinsaver card		1	61.9 (122/197)	[127]
	2014	Whatman^®^ 903 Proteinsaver card		3	96.7 (260/269)	[129]
	2017	Whatman^®^ 903 Proteinsaver card		1	77.0 (114/148)	[123]
	2017	Whatman^®^ 903 Proteinsaver card		1	99.0 (198/200)	[153]
	2020	Whatman^®^ 903 Proteinsaver card		3	46.9 (67/143)	[137]
	2021	Whatman^®^ 903 Proteinsaver card		1	84.4 (27/32)	[122]
	2023	Ahlstrom BioSample Card TFN		1	100.0 (185/185)	[32]
HPV	2008	Whatman^®^ 3MM	Cervicovaginal brush	1	39.1 (36/92)	[97]
	2009	FTA^™^ Elute Micro		1	100.0 (24/24)	[78]
	2010	Whatman^®^ 3MM		2	91.7 (55/60)	[105]
	2011	FTA^™^ Cartridge		2	75.6 (34/45)	[61]
	2013	FTA^™^ Cartridge		2	89.8 (123/137)	[60]
	2015	Biolife Italiana Mascia Brunelli	Urine	1	97.3 (109/112)	[37]
	2015	FTA^™^ Cartridge	Cervicovaginal brush	2	70.7 (70/99)	[59]
	2021	FTA^™^ Indicating Elute	Oral mucosa swab	2	50.0 (30/60)	[23]
IAV	2021	FTA^™^ Classic	Whole blood	5	100.0 (4/4)	[33]
	2021	FTA^™^ Indicating		5	100.0 (4/4)	[33]
	2021	Ahlstrom GenSaver^™^		5	75.0 (3/4)	[33]
	2021	Ahlstrom GenSaver^™^ 2.0		5	100.0 (4/4)	[33]
	2021	Whatman^®^ Human ID Bloodstain		5	75.0 (3/4)	[33]
	2021	Copan Nucleic-Card^™^		5	75.0 (3/4)	[33]
	2021	Macherey-Nagel^™^ Nucleocard^®^		5	100.0 (4/4)	[33]
IBDV	2006	FTA^™^	Bursal tissue impression	1	85.7 (6/7)	[55]
IBV	2005	FTA^™^	Allantoic fluid	5	100.0 (60/60)	[50]
	2006	FTA^™^	Bursal tissue impression	1	88.0 (22/25)	[51]
IHHNV	2025	FTA^™^	Prawn gill tissue	5	100.0 (112/112)	[47]
MDV	2009	FTA^™^	Feather pulp	1	96.2 (50/52)	[40]
	2009	FTA^™^	Whole blood	1	96.7 (42/43)	[40]
	2009	FTA^™^	Tumor	1	100.0 (14/14)	[40]
MeV	2001	Whatman^®^ grade 3	Whole blood	3	47.8 (43/90)	[161]
	2005	Whatman^®^ 903 Proteinsaver card	Oral fluid	1	81.1 (26/32)	[2]
	2012	Whatman^®^ 903 Proteinsaver card		3	100.0 (27/27)	[141]
	2019	FTA^™^ Indicating		1	87.8 (165/188)	[79]
MHV	2021	Allentown^®^ Sentinel^™^ EAD^®^	Rodent bedding	2	50.0 (1/2)	[35]
	2021	Reemay^®^ 2024	Rodent bedding	2	0.0 (0/2)	[35]
MNV	2020	Allentown^®^ Sentinel^™^ EAD^®^	Exhaust air dust	2	98.1 (53/54)	[34]
	2021	Allentown^®^ Sentinel^™^ EAD^®^	Rodent bedding	2	83.3 (10/12)	[35]
	2021	Reemay^®^ 2024		2	50.0 (6/12)	[35]
	2021	Reemay^®^ 2024		2	70.0 (7/10)	[90]
	2023	Reemay^®^ 2024		2	15.9 (10/63)	[89]
NDV	2006	Whatman^®^ grade 1	Allantoic fluid	4	100.0 (7/7)	[159]
	2006	FTA^™^		4	80.0 (4/5)	[53]
	2010	FTA^™^		4	60.0 (3/5)	[52]
	2022	FTA^™^ Classic		4	66.7 (4/6)	[72]
NNV	2016	Whatman^®^ 903 Proteinsaver card	Larval homogenate	1	100.0 (1/1)	[70]
	2016	Whatman^®^ 903 Proteinsaver card	Virus culture	1	100.0 (1/1)	[70]
	2016	Whatman^®^ 903 Proteinsaver card	Milt	1	100.0 (1/1)	[70]
	2016	Whatman^®^ 903 Proteinsaver card	Spiked seaweed	1	100.0 (1/1)	[70]
NoV	2019	Whatman^®^ 3MM	Stool	5	100.0 (12/12)	[99]
	2021	FTA^™^ Micro	Stool	2	77.2 (61/79)	[84]
PPRV	2014	Whatman^®^ 3MM	Whole blood	2	66.7 (12/18)	[98]
PRRSV	1998	Whatman^®^ grade 1	Serum	2	88.9 (128/144)	[25]
	2007	FTA^™^	Whole blood	2	100.0 (6/6)	[9]
	2012	FTA^™^	Serum	1	88.9 (40/45)	[48]
	2012	FTA^™^	Lung	1	100.0 (11/11)	[48]
	2019	FTA^™^	Whole blood	5	66.7 (30/45)	[43]
PUUV	2011	Advantec^®^ Nobuto	Whole blood	1	100.0 (12/12)	[27]
RABV	2003	Whatman^®^ 903 Proteinsaver card	Brain tissue	5	100.0 (50/50)	[150]
	2007	FTA^™^	Virus culture	4	100.0 (4/4)	[54]
	2020	Whatman^®^ 903 Proteinsaver card	Brain tissue	1	96.7 (58/60)	[3]
	2022	FTA^™^ Classic	Brain tissue	3	80.0 (16/20)	[63]
RHDV2	2022	Advantec^®^ Nobuto type II	Whole blood	2	100.0 (77/77)	[31]
RRV	2010	FTA^™^	Mosquito saliva	3	90.0 (27/30)	[44]
RV	2004	Whatman^®^ grade 17	Stool	3	78.8 (63/80)	[160]
	2015	FTA^™^		5	82.5 (38/40)	[36]
	2015	BD BBL^™^ Sensi-Disc^™^		5	82.5 (33/40)	[36]
SARS-CoV-2	2022	FTA^™^ Classic	Nasopharyngeal swab	3	96.8 (122/126)	[66]
	2022	KAJ LAB 0.63 mm thick	Oropharyngeal swab	1	97.2 (35/36)	[87]
	2024	Whatman^®^ 3MM	Saliva	1	74.1 (2121/286)	[106]
SIV	2009	Whatman^®^ 903 Proteinsaver card	Whole blood	5	90.7 (68/75)	[151]
TBEV	2023	Advantec^®^ Nobuto I	Whole blood	1	100.0 (4/4)	[29]
TMEV	2021	Allentown^®^ Sentinel^™^ EAD^®^	Soiled bedding	2	72.7 (8/11)	[35]
	2021	Reemay^®^ 2024	Soiled bedding	2	45.5 (5/11)	[35]
USUV	2022	Whatman^®^ 903 Proteinsaver card	Whole blood	2	53.2 (25/47)	[110]
VEEV	2005	Advantec^®^ qualitative grade 2	Brain homogenate	5	100.0 (10/10)	[26]
WNV	2005	Advantec^®^ qualitative grade 2	Brain homogenate	5	100.0 (10/10)	[26]
	2010	FTA^™^	Mosquito saliva	3	83.3 (25/30)	[44]
	2016	FTA^™^ Micro	Oropharyngeal swab	1	80.6 (25/31)	[82]
	2016	RNASound^®^	Oral swab	1	100.0 (24/24)	[82]
	2019	FTA^™^	Mosquito excreta	3	100.0 (36/36)	[56]
	2021	FTA^™^ Classic	Mosquito saliva	2	100.0 (2/2)	[64]
	2024	Whatman^®^ GB003	Mosquito saliva	3	46.2 (12/26)	[154]
YFV	2005	Advantec^®^ qualitative grade 2	Brain homogenate	5	100.0 (10/10)	[26]
ZIKV	2017	FTA^™^	Mosquitoes	5	100.0 (4/4)	[45]
	2018	Whatman^®^	Mosquito saliva	2	100.0 (9/9)	[95]
	2020	Whatman^®^ 903 Proteinsaver card	Serum	1	100.0 (2/2)	[126]
	2022	Whatman^®^ 903 Proteinsaver card	Whole blood	1	100.0 (2/2)	[115]
